# Vaccinations for Expecting Mothers to Improve Pregnancy Care in Middle Tennessee

**DOI:** 10.3390/pathogens14121255

**Published:** 2025-12-08

**Authors:** Alphonso Harvey, Mohammad Tabatabai, Derek Wilus, Sofia Thomas, James E. K. Hildreth, Donald J. Alcendor

**Affiliations:** 1 Help To Moms, 810 Dominican Way, Nashville, TN 37208-3599, USA; alphonso@helptomoms.com; 2 Office for Research and Innovation, Meharry Medical College, Nashville, TN 37208-3599, USA; 3 Department of Family & Community Medicine, Meharry Medical College, Nashville, TN 37208-3599, USA; 4 Center for AIDS Health Disparities Research, Department of Microbiology, Immunology, and Physiology, School of Medicine, Meharry Medical College, Nashville, TN 37208-3599, USA

**Keywords:** COVID-19, flu, and Tdap vaccination in pregnant women, maternal immunizations, recommended vaccines, Tennessee

## Abstract

**Background:** During pregnancy, mothers and their infants are at increased risk for complications due to COVID-19 infection, influenza, and pertussis. At the time of writing, the previous advisory committee on immunization practices (ACIP) recommended that pregnant women receive the COVID-19 vaccine, influenza, tetanus-toxoid, reduced diphtheria toxoid, and acellular pertussis (Tdap) vaccine, as well as respiratory syncytial virus vaccinations during pregnancy. The COVID-19 pandemic greatly impacted routine vaccinations especially among medically underserved women in the South. The barriers to recommended vaccinations during pregnancy for medically underserved women in the South are unclear and require further investigation. The purpose of this study is to examine the attitudes, opinions, and beliefs of a multiracial pregnant cohort from diverse backgrounds in Central Tennessee about their experiences with the vaccines that are recommended during pregnancy. The vaccines included in the study are COVID-19, flu, and Tdap because RSV was not yet FDA-approved for pregnant women at the launch of this study. **Methods:** In this study, we focus on medically underserved women in Nashville, Tennessee, and the surrounding rural counties regarding vaccine acceptance and initiation of the COVID-19, influenza, and the Tdap vaccines. This study involved 208 pregnant people (100%) aged 18–49 years. All respondents were pregnant at the time of the study. The study consisted of a 26 question Redcap survey about participants’ beliefs, attitudes, opinions, and experiences with the COVID-19, flu, and Tdap vaccines during their pregnancy. **Results:** The randomly selected participants in the cohort were 40.4% White, 31.7% Black, 21.6% Hispanic, and 6.3% other race/ethnicity. The mothers in the cohort were young, with an average age of 27 years, most were married, and 52.8% had an annual household income before taxes of less than USD 35,000. Only 19.2% of the mothers in this study were very confident of the safety of the COVID-19 vaccine, compared to 32.7% for both the flu and Tdap vaccines. Overall, primary care providers were identified as the most trusted messengers for both disease and vaccine information for COVID-19, flu, and Tdap. However, only 11 participants out of 208 received all three of the ACIP recommended vaccines during their pregnancies in the study, barring the time-dependent vaccination for Tdap. The most common reasons for not receiving these vaccines involved concerns for the safety of themselves and their babies and a fear of needles. **Conclusions:** Education and awareness of ACIP-recommended vaccines during pregnancy needs improvement, and the support of primary care providers as the main driver of pregnancy vaccine initiation is essential.

## 1. Introduction

In the USA, it is recommended that all pregnant women receive the COVID-19 vaccine, the seasonal inactivated influenza vaccine, and the Tdap vaccine during the third trimester of pregnancy [1,2,3]. These vaccines can decrease the complications of COVID-19 and risk of flu and pertussis (whooping cough), respectively, among pregnant women and their infants [4] At the time of writing, after the study was initiated on 27 May 2025, the USA Health and Human Services (HHS) Secretary announced that the CDC had removed its recommendation of the COVID-19 vaccine for healthy children and pregnant women [5]. Three days later, on 30 May 2025, the CDC updated its recommendation materials, removing guidance that endorsed use of the vaccines during pregnancy. However, on 22 August 2025, the American College of Obstetricians and Gynecologists (ACOG) continued to recommend updated COVID-19 vaccination for individuals who are pregnant, lactating, or planning pregnancy [6]. Officials of the Food and Drug Administration (FDA), writing in the *New England Journal of Medicine*, recommended that COVID-19 vaccinations be limited to people over 65 as well as those between 6 months and 64 years who have factors that put them at high risk for severe COVID-19 infections [7]. However, prior to this announcement, the head of the Department of Health and Human Services the COVID-19 mRNA vaccines from Pfizer and Moderna were recommended by the FDA for pregnant women and children 6 months of age. In addition, the Centers for Disease Control and Prevention (CDC), American College of Obstetricians and Gynecologists (ACOG), and Society for Maternal-Fetal Medicine (SMFM) recommend the FDA-approved Pfizer and Moderna mRNA COVID-19 vaccines and boosters for all eligible pregnant and lactating women in the USA [8,9,10,11]. Most recently, on September 2023, the FDA-approved Pfizer Respiratory Syncytial Virus (RSV) vaccine was recommended by the ACIP and the CDC for pregnant women at 32–36 weeks gestation using seasonal administration to prevent RSV-associated lower respiratory tract disease (LRTD) in infants aged <6 months [12].

Both observational and prospective studies have shown that the influenza and Tdap vaccines reduce maternal and infant disease burden after vaccination [13,14,15,16]. Maternal immunization with these vaccines can impart immunity towards these diseases to their children who are too young to be immunized. Maternal immunizations are also warranted for emergency travel and under special circumstances to protect mothers and their babies against severe and life-threatening diseases. It is beneficial for pregnant mothers to be vaccinated because their maternal antibodies can cross the placental barrier and impart immunity to their babies [17,18]. It is especially important to protect expecting mothers against COVID-19, influenza, and tetanus.

Disparities in vaccine coverage for expecting mothers in Tennessee and barriers to recommended vaccination warrants further study. Racial and ethnic disparities exist for pregnant African Americans and Hispanic/Latinx women with regard to vaccination against flu and Tdap [19]. African American women have been shown to have lower flu and Tdap vaccination coverage than non-Hispanic White women, while Hispanic/Latinx women have been shown to have higher flu vaccination coverage but lower Tdap coverage than non-Hispanic White women [19]. Lower flu vaccination coverage among medically underserved women have been shown to correlate with women who were less educated, unemployed, living below poverty, living in the South, and without prenatal insurance [19]. Lower Tdap vaccination coverage has been shown to correlate with living below poverty, living in the South, and having public prenatal insurance [19]. The American College of Obstetricians and Gynecologists (AGOG) recommend the FDA-approved Pfizer and Moderna mRNA COVID-19 vaccines and boosters for all eligible pregnant women in the USA [20,21,22]. Unfortunately, underserved pregnant women have been disproportionately impacted by severe COVID-19 disease and COVID-19 vaccine hesitancy [23,24,25]. This has resulted in reduced vaccine uptake of other vaccines among these women [23]. The underlying reasons for poor vaccine uptake among medically underserved pregnant women in Nashville for the COVID-19 vaccines, as well as for other recommended vaccines, are unclear. Previous studies have only examined vaccine access, hesitancy, and uptake of flu, Tdap, or COVID-19 separately of in combination, but not all three together. In our study, we examine COVID, flu, and Tdap together using a more comprehensive survey with fine-tuned options for participants that could afford more detailed observation from this multiracial diverse population. The novelty in this study is that it examines together three of the most important respiratory pathogens that can affect expecting mothers and their babies due to immune downregulation and increased cardiopulmonary load during pregnancy. This study examines both a multiracial and socioeconomic diverse pregnancy cohort in Middle Tennessee where there is a unique mixture of both urban and rural communities. The Redcap survey employed in this study is very comprehensive and goes far beyond traditional racial demographics, vaccine barriers, vaccine confidence, and vaccine uptake. The survey is also written in a manner that supports cultural competency for diverse populations.

This study examines the effects of the COVID-19 pandemic on medically underserved mothers’ access to pregnancy recommended vaccinations. The COVID-19 pandemic greatly impacted routine clinical prenatal visits and scheduled vaccinations for medically underserved pregnant women. Barriers for medically underserved mothers in Nashville to access scheduled COVID-19, influenza, and Tdap vaccines require further investigation.

## 2. Methods

We administered an IRB-approved Redcap survey to mothers via a partnership with HELP TO MOMS, which is a community-based organization that supports expecting and postpartum mothers. The partnership supported vaccine ambassadors that attended maternal events, including baby showers, to recruit participants, who were provided with gift cards to complete the survey. The study was designed to better understand the barriers to vaccine access and vaccine initiation in the different regions of Nashville and the surrounding counties. We aimed to address these barriers, improve vaccine confidence via trusted messengers, and provide mothers with information to address fear and misinformation and provide free direct vaccine access to improve vaccine uptake and support health equity. This study focused on expecting medically underserved mothers, but all of the mothers were invited to participate to make overall comparisons among different racial and ethnic groups. Inclusion criteria were limited to mothers that were 18 years or older, had an uncomplicated pregnancy, and resided in Nashville or the surrounding Central Tennessee counties.

### 2.1. The 15 Tennessee Counties Included in This Study

These counties include Cheatham, Coffee, Davidson, Dickson, Grundy, Hickman, Houston, Humphreys, Maury, Montgomery, Robertson, Rutherford, Warren, Williamson, and Wilson. These counties represent both urban and rural counties in Central Tennessee.

### 2.2. Data Analysis Plan

#### 2.2.1. Power Analysis

Based on our unpublished data, we estimate that 40% of our target subjects are confident in the selected vaccines. We expect our program to increase this proportion to 70%. Based on these statistics, we have determined McNemar’s odds ratio to be approximately 0.538. Using the G*Power software version 3.1.9.7 [26] with a type I error probability of 0.05 and 80% power, we have determined that a two-sided McNemar’s test requires a sample of 225 subjects to participate in our survey both before and after experiencing our program. This also includes an attrition rate of 20%.

#### 2.2.2. Analysis Plan

We will use two methodological approaches to analyze our intervention processes: quantitative analysis using vaccine survey data, and qualitative analysis using data gathered from focus groups and interviews.

#### 2.2.3. Analysis Plan for Quantitative Data

We performed a frequency and exploratory data analysis to detect the presence of potential outliers or influential observations in our data. We compared the data gathered both before and after program experience (engagement with AMB/CHW and MTM partners) for continuous or ordinal variables using the paired/matched *t*-test. In the event of the violation of the normality assumption, we used the non-parametric Wilcoxon signed-rank test. To analyze binary data, we used McNemar’s chi-square test. To analyze categorical variables with three or more categories, we used Cochran’s Q test. Depending on the nature of the outcome variable, we used ordinal or nominal multiple logistic regression to analyze the effect of explanatory variables such as demographic and socioeconomic variables on outcomes such as vaccine hesitancy, confidence, and uptake. We calculated the odds ratios and corresponding 95% confidence intervals. In addition, we validated the results of the multiple logistic regression using hyperbolastic regressions Type I and Type II. We used multiple imputations to address incidences of missing data. We used SAS version 9.4 and SPSS version 28.0.0.0 to conduct all statistical analyses.

#### 2.2.4. Analysis Plan for Qualitative Data

We used constant comparison analysis, classical content analysis, discourse analysis, and micro-interlocutor analysis to analyze the data gathered from focus groups. In addition, we used Venn diagrams to visually present the response patterns of subgroups of interest to each focus group question or across multiple questions. To analyze information from our qualitative interviews, we used both inductive and deductive analyses. In regard to the inductive analysis, we used thematic content analysis and narrative analysis. For the deductive analysis, we built categories and assigned data to these categories.

To measure internal consistency in our survey design, we tested the validity and reliability of our survey using the statistical method of Cronbach’s Alpha. This method indicates how well multiple survey questions or items measure the same underlying concept. The item response analysis for our survey showed a Cronbach Alpha value of 0.861, indicating strong internal consistency, meaning that our survey questions were reliably measuring the same construct. In addition, we carefully checked the correctness in our figures, tables, and terminologies and followed our statistical analysis plan.

## 3. Results

### 3.1. Cohort Demographics

This study included medically underserved populations (MUPs), which refers to groups of individuals who face significant barriers to accessing health care services that can include economic limitations, geographic isolation, cultural or linguistic differences, and discrimination. These populations often have low incomes, lack primary care access, and have high rates of chronic diseases.

**Figure 1:**
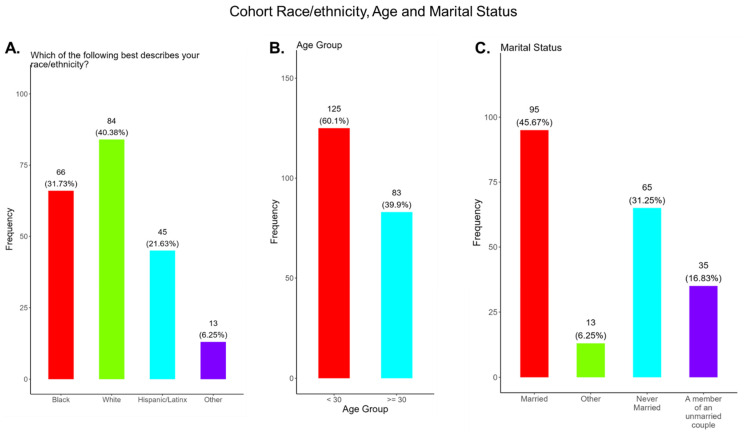
Pregnancy cohort race/ethnicity, age, and marital status.

There were 15 counties in Middle Tennessee with at least one participant in the study. Most participants in the study were from Davidson County (50%), Dickson County (13.5%), Rutherford County (9.1%), Williamson County (4.3%), Sumner County (4.3%) Montgomery County (2.9%), Wilson County (2.4%), Hickman County (1.92%), and Robertson County (1.4%), and all other counties were below 1%. This study was all-inclusive and was not limited to minority women. The cohort consisted of 40.4% White, 31.7% Black, 21.6% Hispanic/Latinx, and 6.3% other race/ethnicity (Figure 1A). Most participants (60.1%) were less than 30 years of age and 39.9% were older than 30 years of age, with an average age of 28 years (Figure 1B). Most participants were married (45.7%) or never married (31.3%), with 16.8% who were members of an unmarried couple (Figure 1C). The remainder of the participants were in other types of relationships.

### 3.2. Differences in Cohort Educational Attainment and Socioeconomic Status

**Figure 2:**
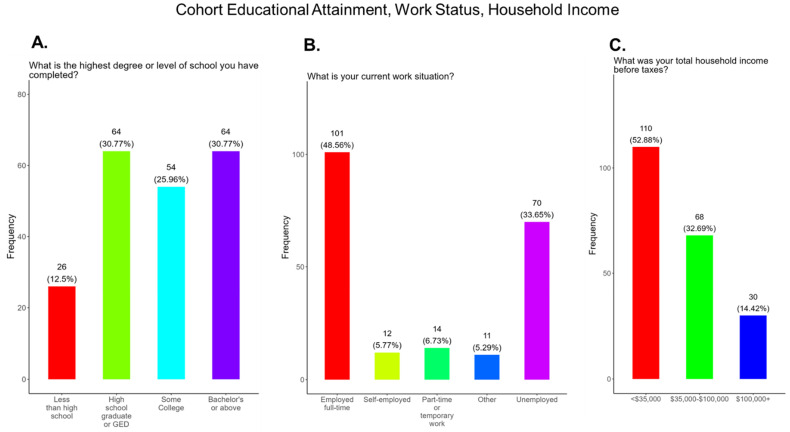
Pregnancy cohort educational attainment, work status, and household income.

In this study, we expected significant differences in cohort education and socioeconomic status. We observed that 12.5% of participants had less than a school education, with 30.8% attaining a high school diploma or GED, 25.9% having some college education, and 30.8% having a bachelor’s degree or above (Figure 2A). We show that 48.6% of participants were fully employed at the time of the survey, 33.7% were unemployed, 5.8% were self-employed, 6.7% were working full-time, and the remainder had other work arrangements (Figure 2B). We observed that 52.9% of participants had household incomes before taxes of <USD 35,000, 32.7% had incomes between 35,000 and 100,000, and 14.4% had incomes of 100,000 or more (Figure 2C).

### 3.3. Strong Supportive Health Coverage and Prenatal Care for Expecting Mothers in Tennessee

**Figure 3:**
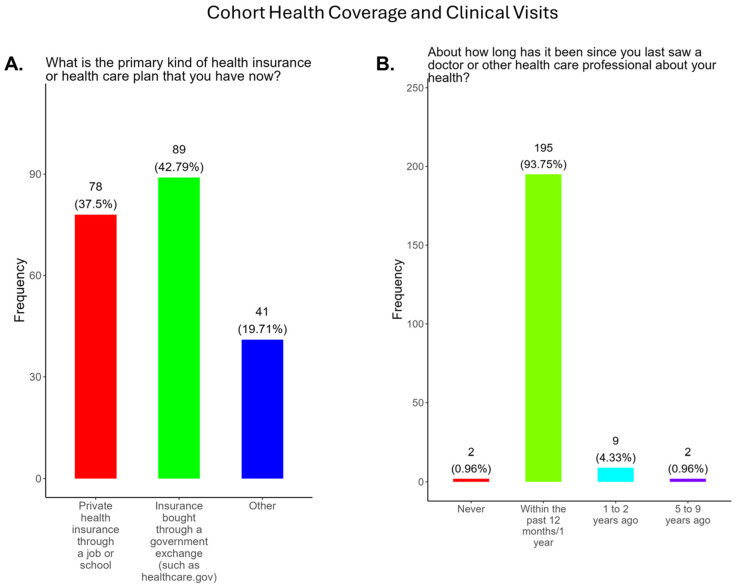
Pregnancy cohort health insurance coverage and clinical visits during pregnancy.

Health insurance and prenatal care are essential to the well-being of mothers and their children. In 2023, approximately 9.35%, or 644,100, Tennesseans were uninsured [27]. The uninsured rate in Tennessee for non-elderly residents is above the USA national average of 9.3%. Hispanic/Latinx communities have the highest uninsured rate (29.4%), followed by Blacks (11.6%). White residents have the second lowest uninsured rate (8.9%), followed by Asian-Americans (8.3%) [28]. In this study, we show that participants had strong support for health coverage in Tennessee. We found that 79.3% of participants had private health insurance through their job or school or insurance purchased through a government exchange program (Figure 3A). Only 19.7% had other forms of insurance (Figure 3A). Recommended prenatal visits with a primary care provider can improve clinical outcomes for mothers and babies. We observed that 93.8% of participants had doctor visits within 12 months and 4.3% and 0.96% visited their physicians within 1–2 years and 5–9 years, respectively (Figure 3A,B).

### 3.4. Poor Confidence in the COVID-19 Vaccine Compared to Flu and Tdap Vaccines

**Figure 4:**
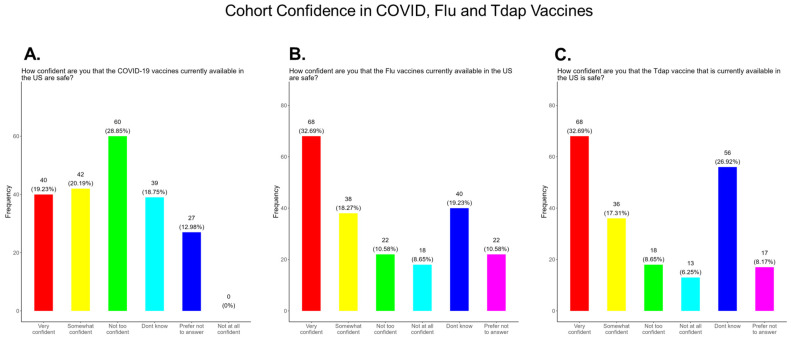
Pregnancy cohort confidence level in COVID, Flu, and Tdap vaccines.

Mistrust and misinformation surrounding the development and dissemination of the COVID-19 vaccines was a driver of vaccine hesitancy that greatly impacted vaccine confidence and uptake in the USA [29,30,31]. Decades of racial injustice and cultural insensitivity experienced by the medically underserved and rural communities fueled COVID-19 vaccine hesitancy, especially in the South, including Tennessee [32]. We observed that only 39.4% of participants in this study were very confident or somewhat confident in the safety of the COVID-19 vaccine compared to 51.0% and 50.0% confident or somewhat confident in the safety of the flu and Tdap vaccines, respectively (Figure 4). Other variations in confidence or no confidence for the different vaccines are shown in Figure 4.

### 3.5. Most Pregnant Women in the Study Were Not Aware That the COVID-19 Vaccine Was Recommended for All Pregnant Women and Were Not Aware of the Health Risks Associated with Being Unvaccinated

**Figure 5:**
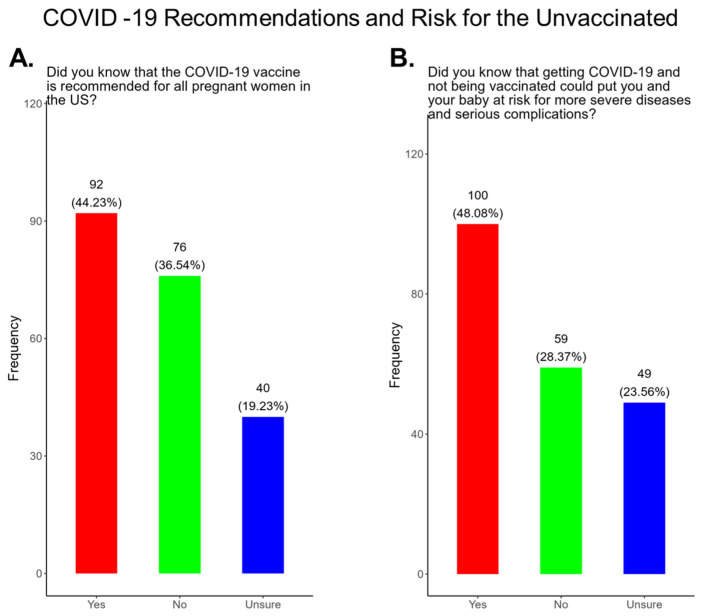
Pregnancy cohort response to COVID-19 recommendations and risk for the unvaccinated.

**Figure 6:**
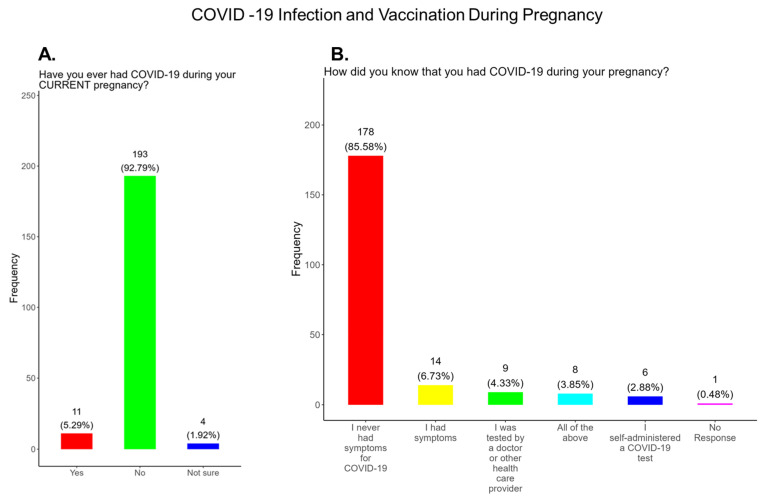
Pregnancy cohort response to COVID-19 infection and vaccination during pregnancy.

The American College of Obstetrics and Gynecology (ACOG) updated its clinical guidance as of 22 August 2025, supporting COVID-19, Flu, Pertussis, and RSV vaccinations during pregnancy [6]. The updated Practice Advisory COVID-19 Vaccination Considerations for Obstetric-Gynecologic Care recommends that patients receive an updated COVID-19 vaccine or “booster” at any point during pregnancy, when planning to become pregnant, in the postpartum period, or when lactating [6]. This study was performed prior to the announcement on 27 May 2025 by the head of the Department of Health and Human Services that stated that COVID-19 vaccines were no longer recommended for pregnant women [5]. It is well documented that the COVID-19 vaccine is effective and safe for women during pregnancy and the infant after birth [32]. It has been shown that antibody persistence in infants was observed when compared to infants whose mother experienced infection during pregnancy without vaccination. Neonatal complications in babies born to mothers infected with COVID-19 that were unvaccinated can include preterm birth, severe neonatal morbidity, medically indicated preterm birth, and severe perinatal morbidity and mortality [32]. We observed that only 44.2% of participants knew that the COVID-19 vaccine was recommended for all pregnant women in the USA, while 55.7% either did not know or were unsure that the COVID-19 vaccine was recommended during pregnancy (Figure 5A). In addition, only 48% of participants knew that receiving COVID-19 and not being vaccinated could put themselves and their babies at risk of complications and severe disease (Figure 5B). In addition, 52.0% of participants did not know or were unsure of the complications and severe disease associated with COVID-19 for them and their babies if the mother became infected and was not vaccinated (Figure 5B). Our study showed that 11 (5.3%) had COVID-19 during their current pregnancy, while 92.8% said that they did not contract COVID-19 during their current pregnancy and 1.9% were unsure (Figure 6A). However, when asked how they knew they had COVID-19, a majority responded that they had symptoms, followed by being tested by a physician and by using a self-administered COVID-19 test (Figure 6B).

### 3.6. Poor Vaccine Confidence and Uptake of the COVID-19 Vaccine for Mothers and Babies and the Need for Health Literacy Surrounding Long COVID-19

Pregnant women were not initially included in COVID-19 vaccine clinical trials, resulting in a lack of information about vaccine safety and pregnancy outcomes compared with the general population [33]. General predictors of COVID-19 vaccine initiation among pregnant women included mothers that were older, were afraid of receiving COVID-19 during pregnancy, had trust in the COVID-19 vaccine, and were defined by a specific race and or ethnicity [34]. However, mistrust in the government, diagnosis of COVID-19 during pregnancy, and fear of side effects and safety concerns were reasons for pregnant women not receiving the COVID-19 vaccination. Uptake of the COVID-19 vaccine by pregnant women in the USA was approximately 27.3% (95% CI: 21.6–33.3%) [35,36] and will require improvement in this population to ensure the protection of mothers at risk and their babies.

**Figure 7:**
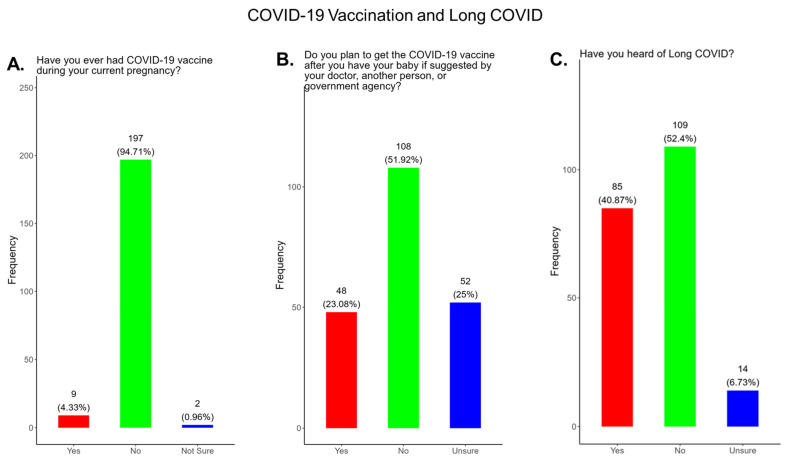
Pregnancy cohort response to COVID-19 vaccination and Long COVID.

In our study, we observed that only 4.3% of participants received a COVID-19 vaccine, while 95.7% did not or were unsure of receiving a COVID-19 vaccination during their current pregnancy (Figure 7A). Only 23% of participants said that they planned to receive a COVID-19 vaccine after delivery if prompted by a physician or government agency, and 72.9% declined receiving a COVID-19 vaccine postpartum (Figure 7B).

A complication of receiving COVID-19 is the development of Long COVID [37]. Studies suggest that women with Long COVID are at higher risk for adverse maternal and neonatal outcomes [38]. The severity of COVID-19 disease during pregnancy and the presence of underlying medical conditions can significantly influence the risk and severity of Long COVID [39]. The incidence of Long COVID among pregnant women varies among the studies performed. In a total of 13 studies performed, with 13,729 participants that involved pregnant women, the prevalence of Long COVID varied widely, ranging from 9.3 to 93% [38]. Prevalence variations are based on differences in study design, follow-up time, and the specific definition of Long COVID-19 used in the study. In a study by Yao et al., pregnant women with long-term COVID-19 had higher risk of developing gestational hypertension [odd ratio (OR) = 3.344], gestational diabetes (OR = 2.301), and fetal intrauterine growth restriction (OR = 2.817) [40].

In our study, when participants were asked if they were familiar with the term Long COVID, only 40.9% said yes and 59.1% said no or were unsure (Figure 7C).

### 3.7. Participant Reasons for and Against Receiving COVID-19 Vaccine and Barriers to Vaccination

**Figure 8:**
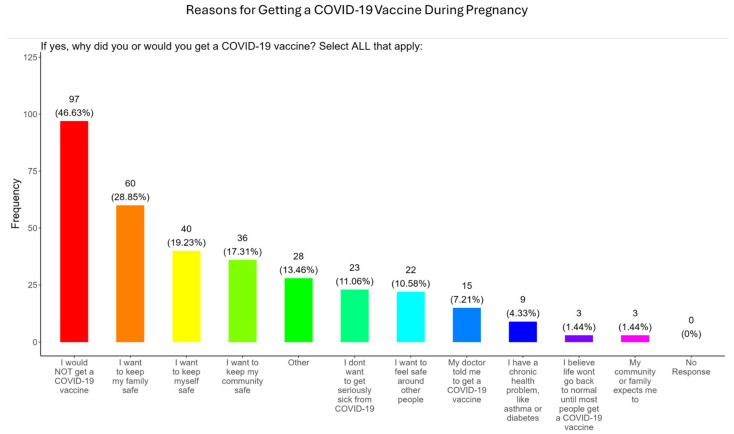
Pregnancy cohort response to receiving a COVID-19 vaccine during pregnancy.

In our study, when participants were asked why did or why they would receive the COVID-19 vaccine during pregnancy, the majority (28.9%) responded that they wanted to keep their family safe, followed by self-safety concerns (19.2%), community safety (17.3%), fear of developing severe COVID disease (11.1%), safety around others (10.6%), advice from a doctor (7.2%), having an existing chronic condition (4.3%), had a need for normal living (1.4%), and were prompted by community or family members (1.4%) (Figure 8). The remainder of participants declined receiving a COVID-19 vaccine (46.6%) or had other reasons for receiving/wanting the vaccine (13.5%) (Figure 8).

**Figure 9:**
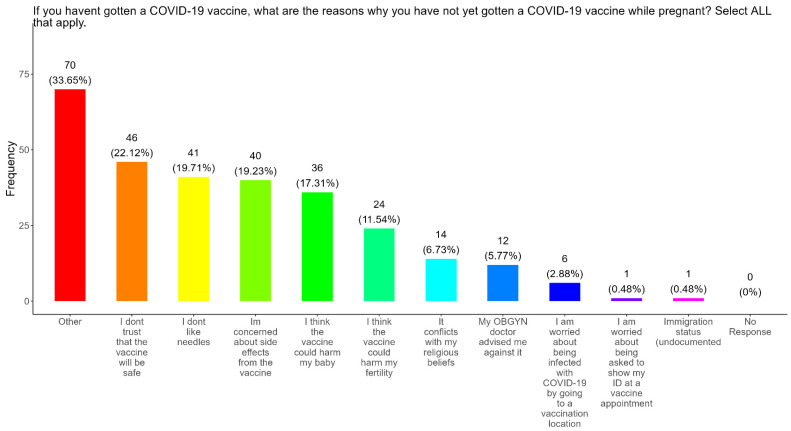
Pregnancy cohort response to Not receiving a COVID-19 vaccine during pregnancy.

When participants were asked why they did not received the COVID-19 vaccine, the majority (22.1%) responded that they did not trust that the vaccines were safe, followed in order by needle phobias (19.7%), concerns about side effects (19.2%), concerns that the vaccine could harm their baby (17.3%), concerns that the vaccine could harm their fertility (11.5%), conflicts with religious beliefs (6.7%), received advice against it by a doctor (5.8%), concerns about being infected with COVID at a vaccination location (2.9%), concerns about showing an ID at a vaccine appointment (0.48%), and concerns about their immigration status being undocumented (0.48%) (Figure 9). The remainder of participants had other reasons for not receiving the COVID-19 vaccine (33.7%) (Figure 9).

**Figure 10:**
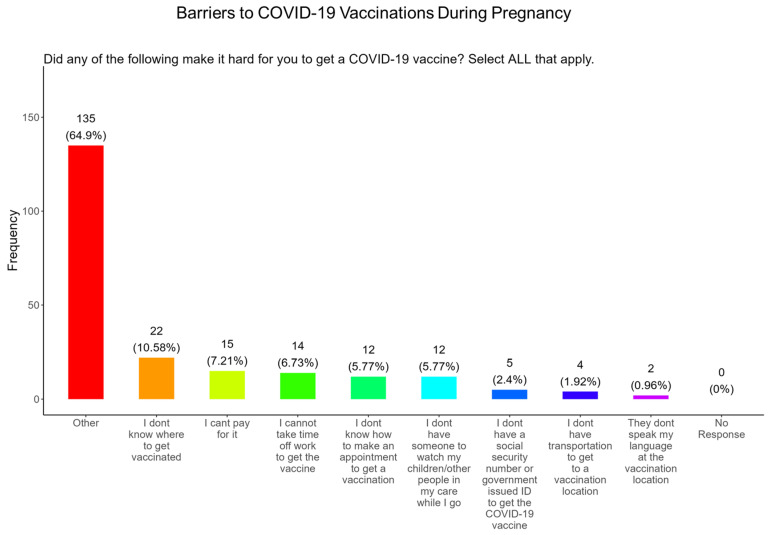
Pregnancy cohort response about barriers to COVID-19 vaccination during pregnancy.

In addition, when participants were asked about the barriers that made it difficult for them receive a COVID-19 vaccine, the majority of participants said that they did not know where to get vaccinated (10.6%) which was followed in order by the following factors: they could not pay for the vaccine (7.2%), could not take time off work (6.7%), did not know how to make an appointment (5.8%), had childcare concerns (5.8%), did not have a social security number or ID number (2.4%), did not have transportation (1.9%), and had language concerns (0.96%) (Figure 10). The remainder of participants had other barriers for not receiving the COVID-19 vaccine (64.9%) (Figure 10).

### 3.8. Study Participants Were Unlikely to Participate in COVID-19 Clinical Trials or Ambassador Programs to Support COVID-19-Related Studies

**Figure 11:**
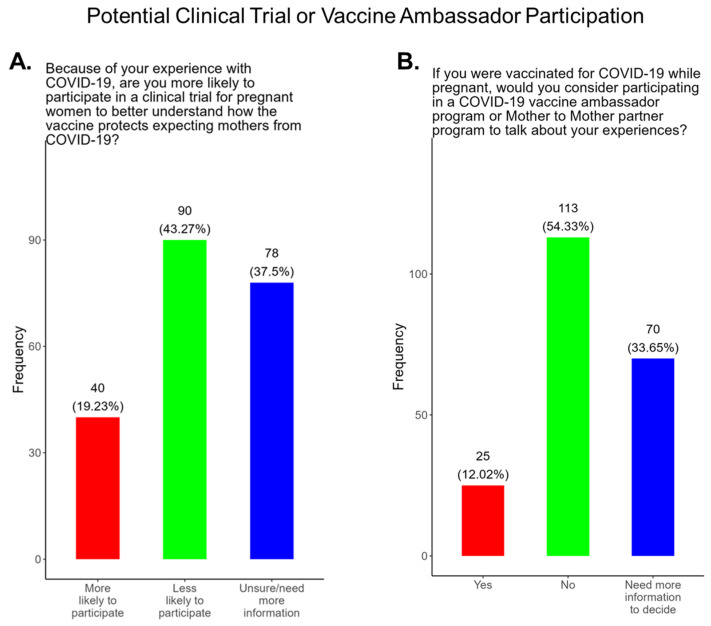
Pregnancy cohort response to possible participation in COVID-19 clinical trials and ambassador programs.

Participants were also asked if they were likely to participate in clinical trials for pregnant women to better understand how the COVID-19 vaccine protects expecting mothers from COVID-19 disease. Most participants (43.3%) responded that they were less likely to participate in a COVID-19 clinical trial, and only 19.2% of participants said that they were more likely to participate in a pregnancy related COVID-19 clinical trial (Figure 11A). The remainder of participants were either unsure or needed more information to decide (Figure 11A). We also asked participants if they would be willing to participate in a COVID-19 ambassador program or Mother To Mother partner program to discuss their experiences with COVID-19 as expecting mothers (Figure 11B). Most participants (54.3%) declined participation in ambassador programs, and only 12.0% were willing to participate (Figure 11B). The remainder of participants (33.7%) needed more information to decide (Figure 11B).

### 3.9. Assessment of the Level of Trust in COVID-19 Information Sources

**Figure 12:**
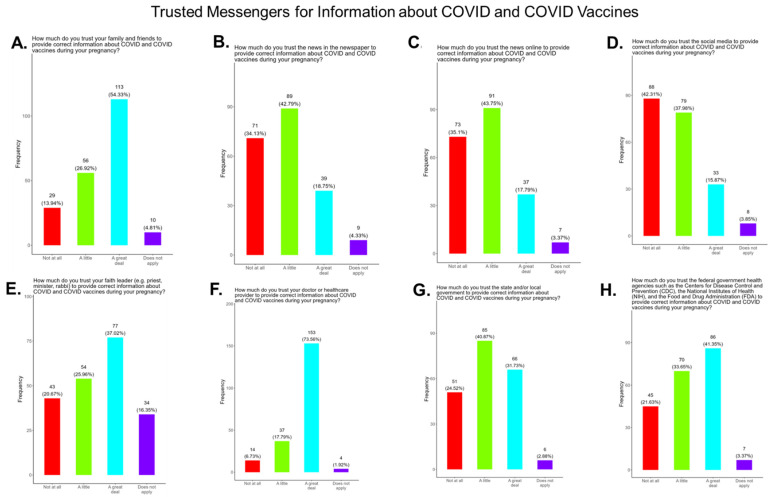
Pregnancy cohort response to questions about trusted messengers for COVID information and COVID-19 vaccines.

Receiving evidence-based information about COVID-19 is essential for expecting mothers to make critical decisions about their health and the health of their babies. We asked the participants which entity they considered to be a reliable source of COVID-19 information based on the options we provided. The participants were also asked to identify their most trusted messengers when needing information about COVID-19 and COVID-19 vaccines. The messengers included family and friends, news and newspapers, online news, social media, faith leaders, primary care providers, local and state governments, and federal government health agencies (CDC, FDA, NIH) (Figure 12). Briefly, for friends and family, 81.3% responded that they were “a great deal” or “a little” reliable sources of COVID information, while 13.94% responded “not at all” and 4.8% responded as “not applicable” (Figure 12A). For news and newspapers, 61.6% responded that they were “a great deal” or “a little” reliable sources of COVID information, while 34.1% responded “not at all” and 4.3% responded as “not applicable” (Figure 12B). For online news, 61.6% of participants responded that they were “a great deal” or “a little” reliable sources of COVID information, while 35.1% responded “not at all” and 3.4% responded as “not applicable” (Figure 12C). For social media, 53.9% responded that they were “a great deal” or “a little” reliable sources of COVID information, while 42.3% responded “not at all” and 3.9% responded as “not applicable” (Figure 12D). For faith leaders, 63.0% responded that they were “a great deal” or “a little” reliable sources of COVID information, while 20.7% responded “not at all” and 16.4% responded as “not applicable” (Figure 12E). For primary care physicians, 91.4% responded that they were “a great deal” or “a little” reliable sources of COVID information, while 6.7% responded “not at all” and 1.9% responded as “not applicable” (Figure 12F). For state and local governments, 72.6% responded that they were “a great deal” or “a little” reliable sources of COVID information, while 24.5% responded “not at all” and 2.9% responded as “not applicable” (Figure 12G). For federal government health agencies, 75.1% responded that they were “a great deal” or “a little” reliable sources of COVID information, while 21.6% responded “not at all” and 3.4% responded as “not applicable” (Figure 12H). Overall, the results show that most participants preferred primary care physicians a little or a great deal as the most reliable sources of information about COVID-19 and COVID-19 vaccines among all of the options presented to the participants (Figure 12F).

### 3.10. Flu Vaccine Initiation, Risk, and Recommendation During Pregnancy

**Figure 13:**
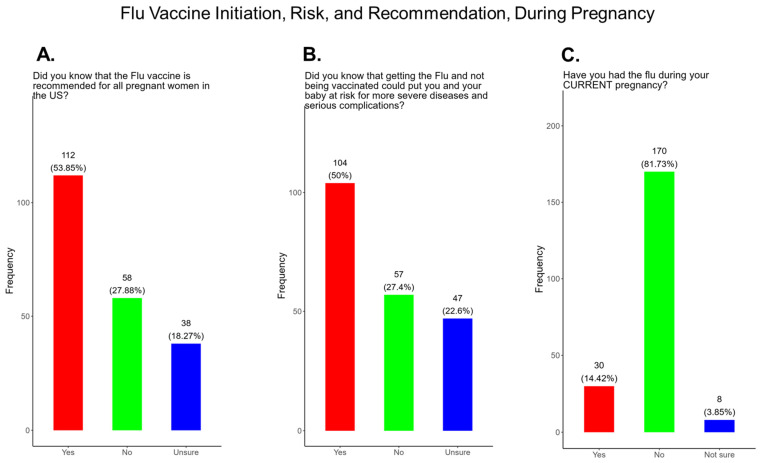
Pregnancy cohort response to flu vaccine initiation, risk, and recommendations during pregnancy.

Flu vaccine uptake among pregnant women in the United States have declined following the COVID-19 pandemic to approximately 38% in March 2025, in contrast to 57.5% flu vaccine coverage among pregnant women during the 2019–2020 flu season [41]. In our study, when participants were asked if the flu vaccine is recommended for all pregnant women in the USA, 53.9% responded yes, 27% responded no, and 18.2% were unsure if they received a flu vaccine during their current pregnancy (Figure 13A). When participants were asked if they knew that receiving the flu and being unvaccinated could lead to severe disease for them and their baby, 50% said yes, 27.4% declined the vaccine, and 22.6% were unsure if they received the flu vaccine (Figure 13B). Having the flu during pregnancy can result in severe complications that can be life-threatening for mothers and their babies. When participants were asked if they contracted flu during their current pregnancy, 14.4% responded yes, 81.7% responded no, and 3.9% of participants were unsure if they had contracted flu during their current pregnancy (Figure 13C).

### 3.11. Family and Personal Safety Were the Most Important Reason for Participants Receiving the Flu Vaccine During Pregnancy

**Figure 14:**
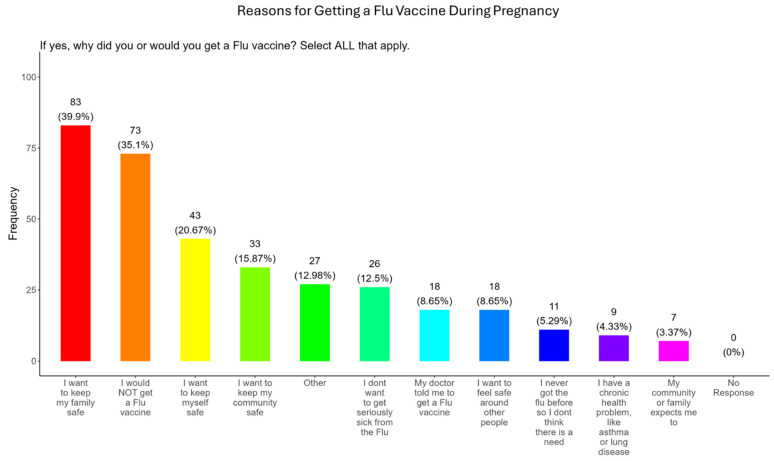
Pregnancy cohort response to receiving a flu vaccine during pregnancy.

In our study, when participants were asked why did or why would they receive the flu vaccine during pregnancy, the majority (40.0%) responded that they wanted to receive the flu vaccine for family and personal safety (21.0%), community safety (15.9%), serious illness from flu (12.5%), because of advice from the doctor (8.7%), safety around others (8.7%), unnecessary because they never had the flu (5.3%), because they had preexisting health conditions (4.3%), and because it was expected by community and family (3.4%) (Figure 14). The remainder of participants declined receiving a COVID-19 vaccine (35.1%) or had other reasons for receiving/wanting the vaccine (13.0%) (Figure 14).

### 3.12. Needle Phobias and Distrust in Vaccine Safety Were the Most Important Reasons for Participants Not Receiving the Flu Vaccine During Pregnancy

**Figure 15:**
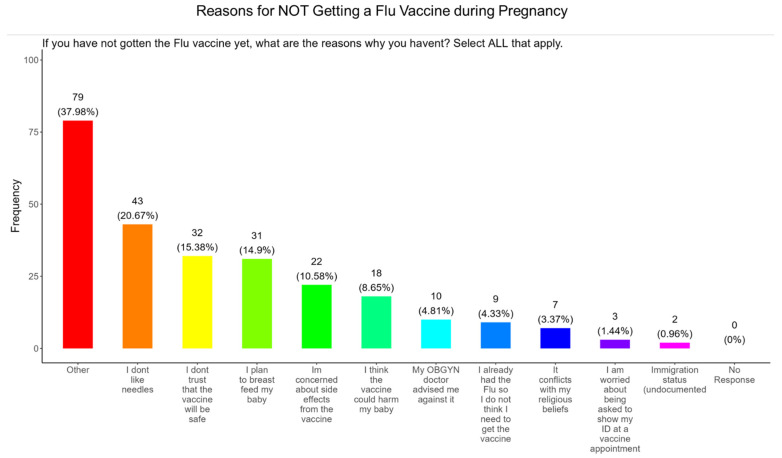
Pregnancy cohort response to not receiving a flu vaccine during pregnancy.

When participants were asked why they did not receive the flu vaccine during pregnancy, the majority (20.7%) responded that they had needle phobias, (15.4%) did not trust the safety of the vaccine, (15.0%) wanted to breastfeed their baby, (10.6%) were concerned about the side effects, (8.7%) were concerned that it would harm the baby, (4.8%) were following advice from a doctor, (4.3%) deemed it unnecessary since they never had the flu, (3.4%) said that it conflicted with their religious beliefs, (1.4%) were concerned about showing identification at the vaccine appointment, and (1.0%) had concerns about immigration status (Figure 15). The remainder of participants declined receiving a flu vaccine for other reasons (38.0%) (Figure 15).

### 3.13. Time off from Work and the Affordability of the Vaccine Were the Most Important Barriers to Participants Receiving a Flu Vaccine During Pregnancy

**Figure 16:**
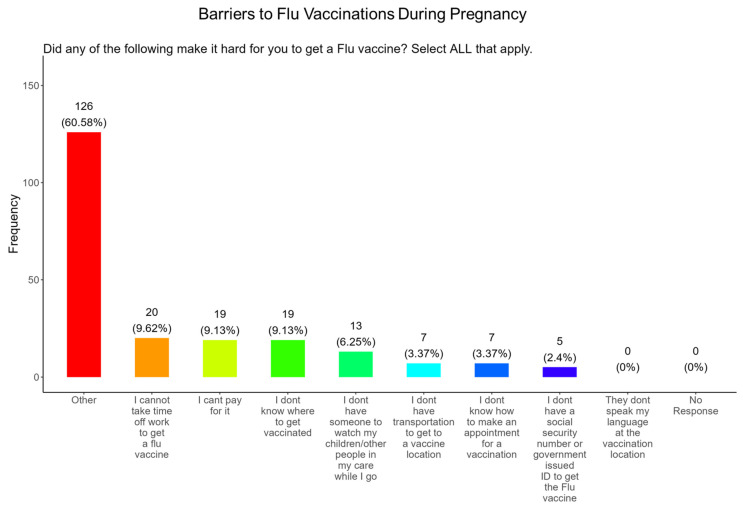
Pregnancy cohort response about barriers to flu vaccination during pregnancy.

When participants were asked what were the barriers to receiving a flu vaccine during pregnancy, the majority (9.6%) responded that they could not take time from work to receive a vaccine, (9.1%) could not afford the vaccine, (9.1%), did not know where to receive vaccinated, (6.3%) were concerned about child care, (3.4%) did not have transportation to the vaccination location, (3.4%) did not know how to make an appointment, and (2.4%) were concerned about not having a social security number or government ID, and there were no concerns about language (Figure 16). The remainder of participants had barriers that were different from the options proposed (60.6%) (Figure 16).

### 3.14. Assessment of the Level of Trust in Flu Information Sources

**Figure 17:**
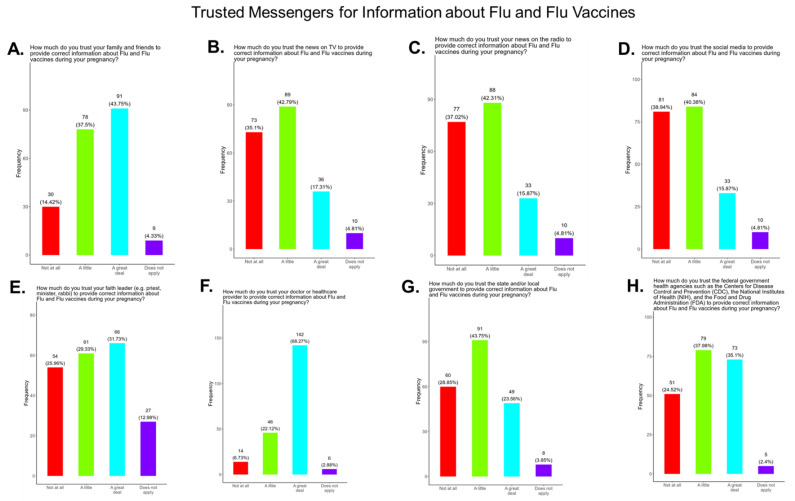
Pregnancy cohort response to questions about trusted messengers for flu information and flu vaccines.

We asked participants to identify their most trusted messengers when needing information about flu and flu vaccines. The messengers included family and friends, news and newspapers, online news, social media, faith leaders, primary care providers, local and state governments, and federal government health agencies (CDC, FDA, NIH) (Figure 17). Briefly, for friends and family, 81.3% responded that they were “a great deal” or “a little” reliable sources for flu information, while 4.4% responded “not at all” and 4.3% responded as “not applicable” (Figure 17A). For news and newspapers, 60.1% responded that they were “a great deal” or “a little” reliable sources of flu information, while 35.1% responded “not at all” and 4.8% responded as “not applicable” (Figure 17B). For news online, 58.3.1% responded that they were “a great deal” or “a little” reliable sources of flu information, while 36.5% responded “not at all” and 5.3% responded as “not applicable” (Figure 17C). For social media, 56.3% responded that they were “a great deal” or “a little” reliable sources for flu information, while 39.0% responded “not at all” and 4.8% responded as “not applicable” (Figure 17D). For faith leaders, 61.0% responded that they were “a great deal” or “a little” reliable sources of flu information, while 26.0% responded “not at” all and 13.0% responded as “not applicable” (Figure 17E). For primary care physicians, 90.4% responded that they were “a great deal” or “a little” reliable sources of flu information, while 6.7% responded “not at all” and 2.9% responded as “not applicable” (Figure 17F). For state and local governments, 67.4% responded that they were “a great deal” or “a little” reliable sources of flu information, while 28.9% responded “not at all” and 3.9% responded as “not applicable” (Figure 17G). For federal government health agencies, 73.1% responded that they were “a great deal” or “a little” reliable sources of flu information, while 24.5% responded “not at all” and 2.4% responded as “not applicable” (Figure 17H).

### 3.15. Only Half of the Expecting Mothers in the Study Were Aware That the Tdap Vaccine Was Recommended for All Pregnant Women, as Well as the Risk of Being Unvaccinated

The tetanus toxoid, reduced diphtheria toxoid, and acellular pertussis (Tdap) vaccine can prevent diphtheria, tetanus, and pertussis (DTP). In 2012, the CDC first began recommending the use of Tdap during pregnancy [19]. Pregnant women are recommended to receive a (Tdap) vaccination during every pregnancy between 27 and 36 weeks to reduce the risk of pertussis to their infants [14]. The Tdap vaccine is proven to be safe and effective for both the mother and baby, and studies have shown that babies are protected from pertussis if the mother is vaccinated during pregnancy [14]. We are aware that, because of variability in time of gestation, some participants were not eligible to receive the Tdap vaccine because it is recommended to be given between 27 and 36 weeks of gestation.

**Figure 18:**
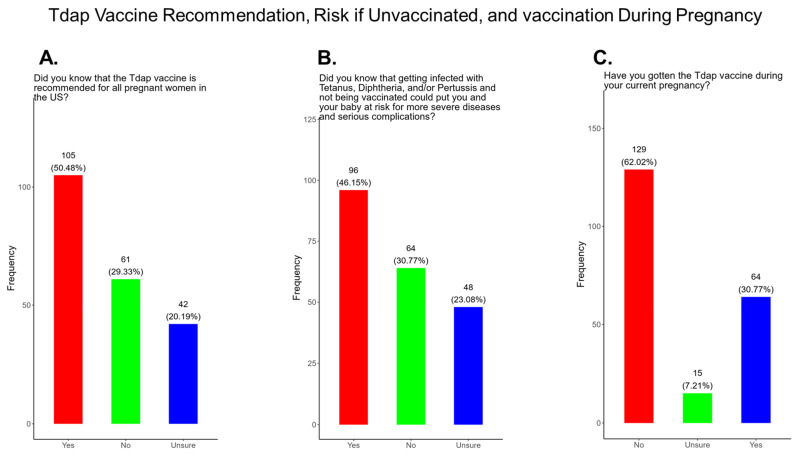
Pregnancy cohort response to Tdap recommendations and risk for the unvaccinated.

In our study, we observed that only 50.5% of participants knew that the Tdap vaccine was recommended for all pregnant women in the USA, while 49.5% either did not know or were unsure that the Tdap vaccine was recommended for all pregnant women in the USA (Figure 18A). In addition, only 46.2% of participants knew that receiving DTP and not being vaccinated could put themselves and their babies at risk of complications and severe disease and 53.9% did not know or were unsure about the risk and complications (Figure 18B). There were 30.8% of participants that received the Tdap vaccine during their current pregnancy, and 62% of participants did not and 7.2% were unsure if they received a Tdap vaccine during their current pregnancy (Figure 18C).

### 3.16. Family and Personal Safety Were the Most Important Reason for Participants Receiving the Tdap Vaccine During Pregnancy

**Figure 19:**
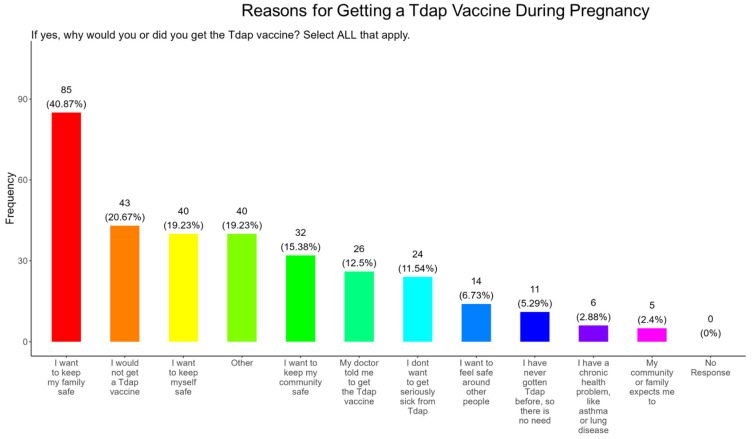
Pregnancy cohort response to receiving a Tdap vaccine during pregnancy.

In our study, when participants were asked why did or why would they receive the Tdap vaccine during pregnancy, the majority (40.9%) responded that they wanted to receive the flu vaccine for family and personal safety (19.2%), followed by community safety (15.4%), advice from a doctor (12.5%), to avoid serious illness (11.5%), safety around others (6.7%), because of never having the DPT (5.3%), having preexisting health conditions (2.9%), and because it was expected by their community and family (4.4%) (Figure 19). The remainder of participants declined receiving a Tdap vaccine (20.7%) or had other reasons for receiving/wanting the vaccine (19.2%) (Figure 19).

### 3.17. Needle Phobias and Plans to Breastfeed Their Infants Were the Most Important Reasons for Participants Not Receiving the Tdap Vaccine During Pregnancy

**Figure 20:**
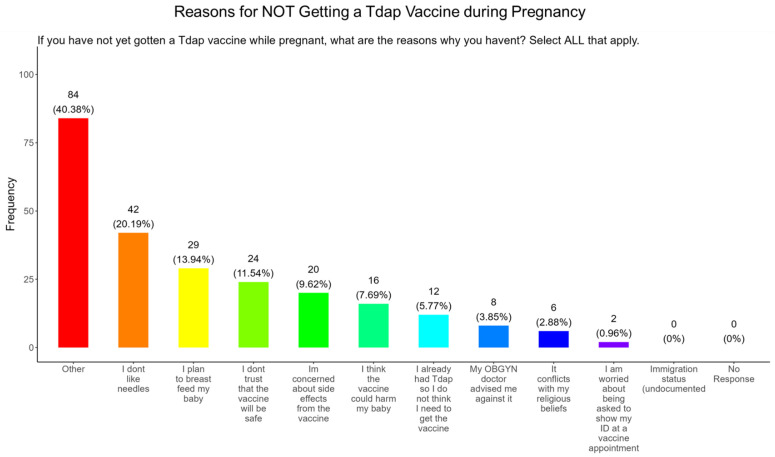
Pregnancy cohort response to not receiving a Tdap vaccine during pregnancy.

When participants were asked why they did not receive the Tdap vaccine during pregnancy, the majority (20.2%) responded that they had needle phobias, (13.9%) wanted to breastfeed their baby, (11.5%) did not trust the safety of the vaccine, (9.6%) had concerns about side effects, (7.7%) were concerned that it would harm the baby, (5.8%) never had the DPT, (3.9%) were following advice from a physician, (2.9%) said it conflicted with their religious beliefs, and (0.96%) were concerned about showing identification at the vaccine appointment. There were no concerns about immigration status (Figure 20). The remainder of participants declined receiving a Tdap vaccine for other reasons (40.4%) (Figure 20).

### 3.18. Vaccine Affordability and Time off from Work Were the Most Important Barriers to Participants Receiving a Tdap Vaccine During Pregnancy

**Figure 21:**
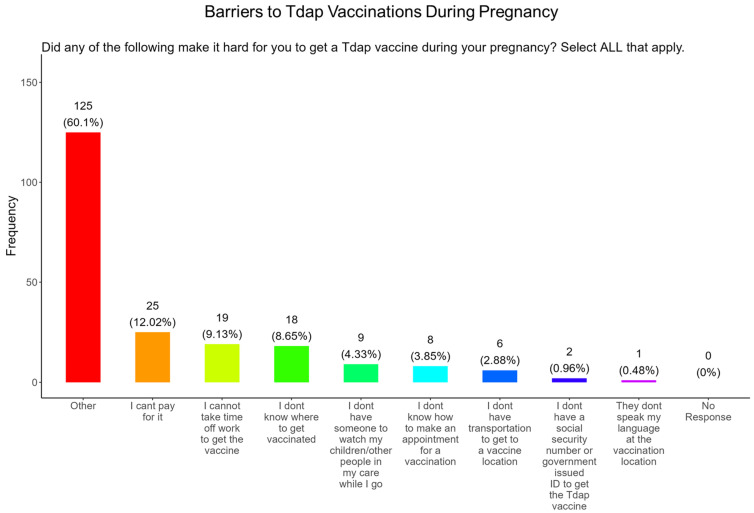
Pregnancy cohort response about barriers to Tdap vaccination during pregnancy.

When participants were asked what were the barriers to receiving a Tdap vaccine during pregnancy, the majority (12.0%) said they could not afford the vaccine, (9.1%) followed by taking time from work to receive a vaccine, (8.7%) did not know where to receive vaccinated, (4.3%) were concerned about child care, (3.9%) did not know how to make an appointment, (2.9%) did not have transportation to the vaccination location, (1.0%) were concerned about not having a social security number or government issued ID, and (0.5%) were concerned about the language at the vaccination location (Figure 21). The remainder of participants had barriers that were different from the options proposed (60.1%) (Figure 21).

### 3.19. Assessing Trust in Tdap Information Sources, Primary Care Providers Were the Most Trusted Source

**Figure 22:**
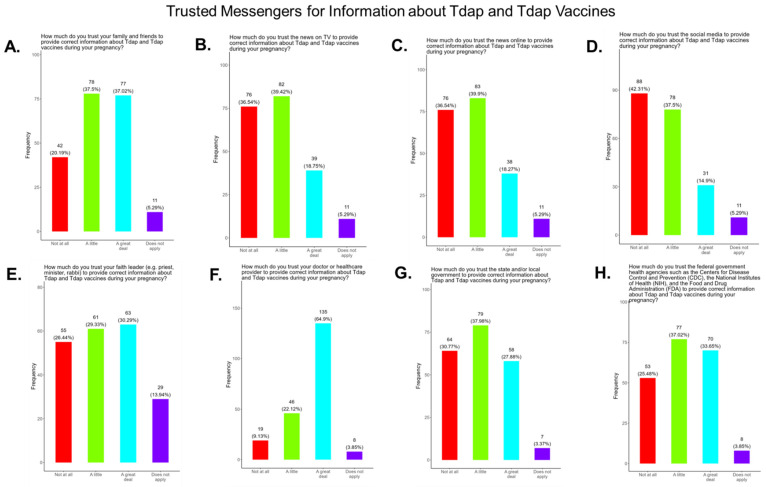
Pregnancy cohort response to questions about trusted messengers for Tdap information and Tdap vaccines.

We asked participants to identify their most trusted source when needing information about Tdap and the Tdap vaccine. The trusted messengers included family and friends, news and newspapers, online news, social media, faith leaders, primary care providers, local and state governments, and federal government health agencies (CDC, FDA, NIH) (Figure 22). Briefly, for friends and family, 74.5% responded that they were “a great deal” or “a little” reliable sources for Tdap information, while 20.2% responded “not at all” and 5.3% responded as “not applicable” (Figure 22A). For news and newspapers, 58.2% responded that they were “a great deal” or “a little” reliable sources of COVID information, while 36.5% responded “not at all” and 5.3% responded as “not applicable” (Figure 22B). For news online, 58.3% responded that they were “a great deal” or “a little” reliable sources of Tdap information, while 36.5% responded “not at all” and 5.3% responded as “not applicable” (Figure 22C). For social media, 52.5% responded that they were “a great deal” or “a little” reliable sources of Tdap information, while 42.3% responded “not at all” and 5.3% responded as “not applicable” (Figure 22D). For faith leaders, 59.6% responded that they were “a great deal” or “a little” reliable sources of Tdap information, while 26.4% responded “not at all” and 14.0% responded as “not applicable” (Figure 22E). For primary care physicians, 87.0% responded that they were “a great deal” or “a little” reliable sources for Tdap information, while 9.1% responded “not at all” and 3.9% responded as “not applicable” (Figure 22F). For state and local governments, 66.0% responded that they were “a great deal” or “a little” as reliable sources for Tdap information, while 30.8% responded “not at all” and 3.4% responded as “not applicable” (Figure 22G). For federal government health agencies, 70.7% responded that they were “a great deal” or “a little” reliable sources Tdap information, while 25.5% responded “not at all” and 3.9% responded as “not applicable” (Figure 22H). Overall, the results show that most participants preferred primary care physicians a little or a great deal as the most reliable source of Tdap information.

## 4. Discussion and Conclusions

This study was conducted in 15 counties, located mainly in Central Tennessee, that represented both urban and rural communities. Most participants were White, Black, or Hispanic/Latinx, with an average age 28 years, and were married (Figure 1). Education attainment among participants varied, with the majority (56.8%) having graduated high school or obtained a GED, along with 31.0% having a bachelor’s degree or above. Most participants were employed either full or part-time; however, 53.0% had a household income of less than USD 35,000 (Figure 2) and, for 2025, the Federal Poverty Level (FPL) for a family of four in the USA is USD 32,150 [42]. All participants had some form of health insurance and a yearly doctor visit that was strongly supported by the State of Tennessee via the TennCare program for expecting mothers (Figure 3). Participants were least confident in the COVID-19 vaccine (19.2%) compared to the flu (33.0%) and Tdap (33.0%) vaccines (Figure 4). The misinformation surrounding vaccine safety, fertility concerns, and overall vaccine hesitancy that plague the development and dissemination of COVID-19 vaccines is the likely reason for this finding.

**The COVID-19 vaccine:** Most participants were aware that the COVID-19 vaccine was recommended for all pregnant in the USA (Figure 5). However, a significant number did not know or were unsure which represents a knowledge/awareness gap in COVID-19 vaccine health literacy that should be addressed targeting under-resourced mothers living near the federal poverty level in Central Tennessee (Figure 5). Most participants (48.0%) were aware of the complications of severe disease themselves and their baby if infected with COVID and being unvaccinated (Figure 5). However, 52.0% were not aware of disease complications or were unsure, which represents a knowledge gap that should be addressed (Figure 5). When participants were asked if they had COVID-19 during pregnancy, 5.3% said yes and 94.7% said no or were unsure (Figure 6). However, when asked how they knew that they contracted COVID, it was unclear because their responses were not consistent with a confirmatory test for diagnosis of COVID-19 (Figure 6). It is essential that COVID-19 disease be diagnosed by a physician/health care provider or with a self-administered COVID-19 test. In our study, COVID-19 vaccine uptake among participants was 4.3%, 95% declined the vaccine, and only 1% were unsure if they received the vaccine (Figure 7). Poor uptake of the COVID-19 vaccines, especially among pregnant women, needs to be addressed now that the Department of Health and Human Services secretary has terminated their recommendation of the COVID-19 vaccines and boosters for all pregnant women, putting the health of expecting mothers and their babies at risk. When participants were asked if they plan to receive the COVID-19 vaccine after they have their baby if suggested by a doctor or a government agency, 23.1% said yes and 77% said no or were unsure (Figure 7). This would suggest that barriers to receiving the COVID-19 vaccine are beyond pregnancy, and participants have concerns postpartum. Although pregnant women are at increased risk for developing Long COVID, the majority (52.4%) of participants in the study had never heard of Long COVID (Figure 7). In a new study supported by the NIH Researching COVID to Enhance Recovery (RECOVER) initiative found that 9.3% of study participants who had COVID-19 during pregnancy went on to experience long-term symptoms [43]. Investigators assessed 1500 pregnant women in the USA that self-reported Long COVID symptoms at least six months post-infection [43]. Therefore, pregnant women must be informed to identify the lingering symptoms associated with Long COVID to assist physicians in diagnosis. Participants’ family and personal safety were the most important reasons for receiving the COVID-19 vaccine. Now that the HHS has terminated COVID-19 vaccine recommendations for pregnant women, this could result in limited vaccine access and increased vaccine hesitancy in this population, leading to a further decline in vaccine coverage during pregnancy. When participants were asked about their reasons for receiving the COVID-19 vaccine during their pregnancy, the majority responded that it was for family and personal safety (Figure 8). When participants were asked about their reasons for not receiving the COVID-19 vaccine during their pregnancy, the majority responded that they did not trust the safety of the COVID-19 vaccine and that they have a needle phobia (Figure 9). COVID-19 vaccine safety concerns have consistently been the most important reason for vaccine hesitancy and poor vaccine uptake, which is driven by misinformation. The important barriers to vaccine compliance, as identified by the participants, are not knowing where to receive the vaccines and vaccine affordability (Figure 10). Participants should be aware that the COVID-19 vaccines are available free of charge at their local health departments, available at no charge via voucher programs through local pharmacies, and without a copay via most state and private insurance providers. Participants were asked if they were likely to participate in COVID-19-related clinical trials or ambassador programs to support COVID-19 vaccine research in pregnancy, and only 19.2% and 12.0% would likely participate in clinical trials or ambassador programs, respectively (Figure 11). Poor participation rates of minority women in clinical trials have been a longstanding problem, contributing to health inequities, and this needs to be addressed [44]. Longstanding barriers to clinical trial access for minority women include mistrust, racial bias, cultural barriers, lack of awareness and education about clinical trials, lack of outreach programs, trial centers not situated in appropriate geographic locations, and low referrals by primary care providers [45,46]. Strategies to improve clinical trials access for minority women should focus on circumventing these barriers. When asked about trusted messengers for COVID-19 and COVD-19 vaccines, participants identified doctors and health care providers (as measured by a great deal + a little) as the most trusted messengers (91.4%), followed in order by friends and family (81.3%), government health agencies (75.1%), state and local government (72.6%), faith leaders (63.0%), and a tie between news and newspapers and online news, both at 61.6% (Figure 12). This finding is consistent with our previous studies [23]. Health care providers know the medical history of their patients and have established trust over an extended period, and they often engage patients regularly by appointment, and there is a sharing of confidential information that reinforces trust. Pregnant women are at higher risk of severe disease if infected with flu and they are unvaccinated. However, 55.8% of participants did know or were unsure that the COVID-19 vaccine was recommended for all pregnant women in the USA (Figure 5).

**Flu vaccine:** Most participants (53.9%) were aware that the flu vaccine is recommended for all pregnant women in the USA (Figure 13). However, 46.2% of participants were not aware of or were unsure of this recommendation. In addition, only half of participants knew that receiving the flu and not being vaccinated could result in severe disease and complications, and 14.4% were infected with flu during their pregnancy (Figure 13). It is essential that the most trusted messengers for health information about flu, that includes primary care providers, health care workers, and dulas, provide information to their pregnant patients about the risk associated with being infected with flu and not being vaccinated. However, receiving vaccinations is a personal choice, and our study reveals the concern participants have for the safety of their babies. When participants were asked about their reasons for receiving a flu vaccine during their pregnancy, the majority responded that it was for family and personal safety (Figure 14). This finding is consistent within this study. When participants were asked about their reasons for not receiving a flu vaccine during their pregnancy, the majority responded that they had a needle phobia and did not trust the safety of the flu vaccine (Figure 15). It was surprising to find that needle phobias were the most important reason for participants to decline a flu vaccination. However, needle phobia, or trypanophobia, is a significant reason for declining vaccinations. This condition is more prominent in children and adolescents and can disrupt or prevent a person from receiving the recommended vaccinations. Trypanophobics can develop intense fear that can trigger a vasovagal response, leading to an abrupt drop in blood pressure and syncope [47,48]. Pregnancy outcomes in women with severe needle phobia can result in the refusal of essential medical care because of fear. However, in a study by McAllister et al., pregnancy outcomes in women with severe needle phobia were not clinically significant. When participants were asked about barriers for not receiving a flu vaccine during their pregnancy, the majority responded that they could not take time off from work to receive the vaccine and they could not afford to pay for the vaccine (Figure 16). These findings could be associated with the socioeconomic status of most participants in which 53% had incomes below USD 35,000 a year, which can make it difficult to afford childcare and to pay out of pocket for vaccines (Figure 2C). However, flu vaccines can be free of charge if participants have transportation to vaccination locations. When participants were asked for their most trusted sources of information about flu and flu vaccines, they preferred primary care physicians (90.4%) a little or a great deal as the most reliable source of information about flu and flu vaccines among all options presented to participants (Figure 17F). The selection of physicians for the most sources of flu information was followed in order by friends and family (81.3%), federal government health agencies (73.1%), state and local governments (67.4%), faith leaders, news and television were equal at (60.1%), news and radio (58.2%), and, finally, social media (56.3%).

**Tdap Vaccine:** Most participants were aware that the Tdap vaccine was recommended for all pregnant women in the USA (Figure 18). However, a significant number (49.5%) did not know or were unsure of the Tdap recommendation (Figure 18). In addition, a majority (53.9%) of participants did not know or were unsure of the infection with DPT and being unvaccinated could result in severe disease and complications for themselves and their babies (Figure 18). Only 30.8% of participants in this study were sure they received the Tdap during pregnancy, and 62.0% were sure that they did not receive the Tdap vaccine. Again, we are aware that the timeline to receive the Tdap vaccine is between 27 and 36 weeks of gestation and many of the participants in this study did not meet these criteria for receiving a Tdap vaccination (Figure 18). When participants were asked about their reasons for receiving a Tdap vaccine during their pregnancy, the majority responded that it was because of family and personal safety (Figure 19). This finding is also consistent with what we observed in this study with the COVID-19 vaccine. When participants were asked about their reasons for not receiving a Tdap vaccine during their pregnancy, the majority responded that it was because of a needle phobia and plans to breastfeed their infant. Participants (14%) were not aware that it is safe to receive the Tdap vaccine if they plan to breastfeed their babies (Figure 20). The ACOG recommends that pregnant women to receive the Tdap vaccine, even if they plan to breastfeed their baby [49]. The vaccine may also provide benefits to breastfed infants via the passing of maternal antibodies to the baby in breast milk that can help protect against pertussis [50,51,52,53]. The Tdap vaccine may also be administered postpartum to women who were not vaccinated during pregnancy [54]. When participants were asked about the barriers to receiving a Tdap vaccination, the majority responded that they could not afford the vaccine and that they could not take off time from work to get vaccinated (Figure 21). Finally, when asked about trusted messengers for DPT and the Tdap vaccine, participants identified doctors and health care providers (measured as “a great deal” + “a little”) as the most trusted messengers (87.0%), followed in order by friends and family (75.0%), government health agencies (70.7%), state and local government (65.9%), faith leaders (59.6%), news online (58.3%), news on television (58.2%), and finally social media (53.0%) (Figure 22). Although the medically underserved population can be distrusting of the medical community, they identified doctors and health care providers as being the most trusted messengers for information about COVID-19, flu, and Tdap. It is therefore critical that health care providers continue to promote evidence-based information about all recommended vaccines to ensure the health and safety of their patients.

## 5. Analysis of Study Findings with Previous Studies

In a recent cross-sectional survey study by Dancewicz et al., they examined the attitudes of 662 pregnant or breastfeeding Polish women towards COVID-19 vaccinations from 20 April 2021 to 23 October 2021 using an online questionnaire distributed via social media [55]. The results show that only one third of participants wanted to be vaccinated against COVID-19; medical professionals with higher education that had trust in their doctors were likely to accept a COVID vaccination and felt that the vaccine was effective. In addition, women that were afraid of receiving COVID were more likely to accept vaccination [55]. We also observed that personal safety and safety of family and friends motivated participants to vaccinate against COVID-19. In our study, acceptance of the COVID-19 vaccine among participants was only 4.3%. This would suggest that vaccine health literacy could improve COVID-19 vaccine uptake among diverse populations. In another recent study by Mierzwa et al., they examined participants’ knowledge and attitudes toward pertussis, influenza, and COVID-19 and their motivations for receiving vaccinated during pregnancy [56]. This study was performed using an anonymous, self-reported questionnaire developed for this study and was distributed to postpartum women that were hospitalized between February and April 2023. Participants were asked to provide sociodemographic and obstetric information, reasons for receiving vaccinated or not, and their sources of vaccination knowledge. The results show that participants’ primary reason for accepting vaccinations was to protect their children from severe diseases associated with pertussis, influenza, or COVID-19. The primary reason why participants declined the vaccines was the belief that the vaccine was unnecessary. The results also show that 27.6% of participants had received the vaccine before pregnancy. Only 49.3% of obstetricians provided information to participants about recommended vaccination during pregnancy. However, 64.2% of surveyed patients expressed a willingness to vaccinate their children with mandatory and recommended vaccines. Our study only examined women that were currently pregnant, and our survey instrument was a comprehensive recap survey about COVID, flu, and Tdap vaccines. Participants in our study cited lack of trust and the fear needles as the primary reasons for declining the vaccines. Again, these findings suggest that vaccine health literacy needs to be improved in this population. A study by Cubizolles et al. examined intentions to vaccinate against influenza, COVID-19, and pertussis and to receive a future vaccine against respiratory syncytial virus in pregnant women [57]. This study was a cross-sectional survey of pregnant women at a University Hospital in France, assessing their knowledge and attitudes toward vaccination against influenza, COVID-19, Tdap, and RSV during pregnancy. There were 310 participants that completed a questionnaire that revealed intentions to become vaccinated were, respectively, 43.9%, 36.8%, 36.1%, and 39.4% against influenza, COVID-19, pertussis, and RSV. The results show that vaccine acceptance was associated with confidence for influenza, COVID-19, and RSV vaccines. Even more flu vaccine acceptance was strongly associated with recommendations from a physician. Lastly, they found that recent vaccination against Pertussis was not a barrier to Pertussis vaccination. In our study, we also find that physicians recommendations are a positive driver of vaccine initiation.

## 6. Limitations of the Study

The limitations of this study include that most surveys were administered in Davidson County, Nashville, in Middle Tennessee. The manuscript was written prior to the HHS Secretary announcement of the withdrawal of the CDC’ general recommendation for routine COVID-19 vaccination during pregnancy. Receiving the Tdap vaccine during pregnancy is gestation-dependent and would influence the responses by participants who were asked if they received a Tdap vaccine during their pregnancy. The level of influence of misinformation among participants is unclear in the survey. The survey allowed for participants to select all options that apply, and it is plausible that some of the respondents had overlap with some of their responses.

## 7. Future Directions

Authors’ response: We agree with the reviewer and have added the following information and additional references to the revised manuscript in blue text. Recommended vaccinations during pregnancy may provide additional maternal/fetal protections surrounding modulations in oxidative stress during pregnancy that can be induced by respiratory viral infections [58]. These future studies, via collaboration, could involve the collection of oxidative stress biomarkers from expecting mothers that were vaccinated for COVID, flu, or Tdap compared those that decline one more of these vaccines. These biomarkers could vary to include lipid peroxidation markers (F2-isoprostanes or their urinary metabolites), DNA oxidation (8-OHdG), protein oxidation (protein carbonyls, nitrotyrosine), redox status (GSH/GSSG), and human serum albumin Cys34-based metrics such as total free thiol or adductomics [59,60,61,62]. Oxidative stress in pregnancy induced by vaccine-preventable respiratory pathogens such as COVID-19, flu, and pertussis have been linked to adverse pregnancy outcomes. Placental and systemic oxidative stress in unvaccinated pregnant women has been implicated in complications such as preeclampsia, fetal growth restriction, premature rupture of membranes, gestational diabetes mellitus, and recurrent pregnancy loss [63,64,65,66]. Therefore, recommended vaccinations to protect against these pathogens could provide added benefits by reducing oxidative stress levels during infection to support the health and wellness of mothers and their babies. Our findings of validated attitudinal measures in this study, paired with maternal or cord-blood oxidative stress biomarkers in a mixed-methods design, would provide strong support for pregnancy-recommended vaccines that should improve vaccine uptake.

## Data Availability

This manuscript did not report any laboratory-based data.
